# Species distribution models predict genetic isolation of *Hetaerina vulnerata* Hagen in Selys, 1853 (Odonata, Calopterygidae)

**DOI:** 10.1002/ece3.70107

**Published:** 2024-08-06

**Authors:** Austin R. Biddy, Joseph D. Manthey, Jessica L. Ware, Nancy E. McIntyre

**Affiliations:** ^1^ Department of Biological Sciences Texas Tech University Lubbock Texas USA; ^2^ Department of Biology University of Alabama at Birmingham Birmingham Alabama USA; ^3^ American Museum of Natural History New York New York USA; ^4^ Present address: Department of Biology University of Alabama at Birmingham Birmingham Alabama USA

**Keywords:** bioclimatic niche, Canyon Rubyspot, Grinnellian niche, landscape genetics, SDMs

## Abstract

Understanding how past and current environmental conditions shape the demographic and genetic distributions of organisms facilitates our predictions of how future environmental patterns may affect populations. The Canyon Rubyspot damselfly (Odonata: Zygoptera: *Hetaerina vulnerata*) is an insect with a range distribution from Colombia to the arid southwestern United States, where it inhabits shaded mountain streams in the arid southwestern United States. Past spatial fragmentation of habitat and limited dispersal capacity of *H. vulnerata* may cause population isolation and genetic differentiation, and projected climate change may exacerbate isolation by further restricting the species' distribution. We constructed species distribution models (SDMs) based on occurrences of *H. vulnerata* and environmental variables characterizing the species' niche. We inferred seven current potential population clusters isolated by unsuitable habitat. Paleoclimate models indicated habitat contiguity in past conditions; projected models indicated some habitat fragmentation in future scenarios. Seventy‐eight *H. vulnerata* individuals from six of the current clusters were sequenced via ddRADseq and processed with Stacks. Principal components and phylogeographic analyses resolved three subpopulations; *Structure* resolved four subpopulations. *F*_ST_ values were low (<0.05) for nearby populations and >0.15 for populations separated by expanses of unsuitable habitat. Isolation by distance was an existing but weak factor in determining genomic structure; isolation by environment and the intervening landscape explained a significant proportion of genetic distance. *Hetaerina vulnerata* populations were shown to be isolated by a lack of tree canopy coverage, an important habitat predictor for oviposition and territoriality. Thus, *H. vulnerata* populations are likely separated and are genetically isolated. Integrating SDMs with landscape genetics allowed us to identify populations separated by distance and unsuitable habitat, explaining population genetic patterns and probable fates for populations under future climate scenarios.

## Graphical abstract

We used historical, current, and future climate species distribution models (SDMs), as well as SDMs with climate and habitat variables, to predict the genetic diversity, differentiation, and clustering of a high‐altitude, stream specialist damselfly (*Hetaerina vulnerata*) in the southwestern United States. With ddRADseq data, we uncovered 3–4 populations of *H. vulnerata* across the landscape. With habitat SDMs and isolation by environment models, we determined that tree coverage is impeding gene flow, and it is an important predictor for this species' ecology (e.g., territoriality, oviposition, and thermoregulation).

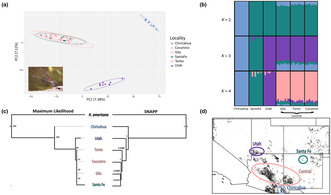

## 1 INTRODUCTION

Aquatic ecosystems at high altitudes (>1500 m above sea level) are isolated habitats surrounded by lower elevation areas that are typically drier in xeric regions. These ecosystems and the populations they support are anticipated to experience unprecedented warming, evaporation, and loss of habitat resources in the future (Beever et al., 2010; Birrell et al., 2020; Diaz et al., 2014; Miller et al., 2021; Ohmura, 2012; Peeters et al., 2002; Urbani et al., 2017). The southwestern United States in particular has a high vulnerability to climatic change that is compounded by the inherent aridity of the region (Chylek et al., 2014; Yanahan & Moore, 2019). The southwestern United States has experienced increasing temperatures over the last several decades (estimated at +0.45°C since the mid‐20th century; Van Devender & Brusca, 2015). Future climatic models suggest that increased temperatures, moderately decreased precipitation, and increased drought frequency are likely in the southwestern United States by the end of the twenty‐first century (Miller et al., 2021; Van Devender & Brusca, 2015), which may have genetic ramifications on organisms in this region through isolation by environment (IBE).

Species' present‐day distributions and genetic variation may reflect environmental conditions from the Last Glacial Maximum (LGM; ~22,000 years ago) and Mid‐Holocene (MH; ~6000 years ago). Organisms expanding along a northern latitudinal gradient are expected to exhibit a pattern of lower heterozygosity in northern populations because of founder effects, lack of gene flow, and relatively smaller effective population sizes (Hampe & Petit, 2005). Therefore, organisms that occupied refugia during glacial cycles should have a different genetic composition from populations that dispersed to available habitat after temperatures became more optimal (Graham et al., 2020), as gene flow was likely severed between populations due to postglacial isolation, especially at high elevations. Since climate change may potentially exacerbate spatial and genetic isolation, it is imperative to quantify past, current, and future habitat suitability, and species distribution models (SDMs) are a commonly used approach to examine shifts in suitability over time (Beck, 2013; Elith & Leathwick, 2009; Urbani et al., 2017; Zhu et al., 2013). Projected SDMs demonstrate vulnerability of aquatic species in xeric areas of the southwestern United States and raise concerns about range truncations and the possibility of extirpation (Yanahan & Moore, 2019). By combining paleoclimate, present climate, and future climatic SDMs, potential locations of organisms can be modeled to provide insight into how shifting distributions have generated contemporary populations and genetic patterns. SDMs have been applied to almost all taxonomic groups, but the ecological and taxonomic diversity of insects makes them flagships for examining how climate has shaped distributional patterns.

As ectotherms, insects are inherently susceptible to fluctuations in temperature and water availability, and lotic specialists with somewhat limited dispersal abilities within mountain streams in otherwise xeric areas often exhibit genetic structuring (Abernethy et al., 2022; Johnson, 1973; Múrria et al., 2019; Phillipsen et al., 2015). Odonata (dragonflies and damselflies) have aquatic egg and juvenile (naiad/nymph) stages and an aerial/terrestrial adult stage. Because of this amphibious life history, odonates are particularly sensitive to habitat changes and drought (Angert et al., 2011; Bybee et al., 2016; Hassall, 2015); indeed, odonates are considered an important group of wetland indicators (Oertli, 2008). Habitat specificity compounded with habitat isolation suggests that odonates may be prone to population isolation and impacted by climate change (Bellis et al., 2021; Bush et al., 2014). Damselflies (suborder Zygoptera) in particular should be excellent candidates for studying how genetic structuring may be related to habitat isolation because of their relatively low vagility and high habitat specificity (Lorenzo‐Carballa et al., 2015).

Several studies employing SDMs have indicated projected climate change effects on odonates, including geographic isolation, hindered dispersal by loss of habitat, and reduced habitat suitability (Amundrud et al., 2017; Bellis et al., 2021; Bush et al., 2014; Collins & McIntyre, 2017). However, few studies have examined the role of paleoclimate on genetic patterns in Odonata (Xue et al., 2017) or how past environmental conditions may explain current genetic patterns (Goodman et al., 2023). Although SDMs map landscape and habitat suitability, they can also be used to infer potential gene flow between populations (Park et al., 2021).

**FIGURE 1:**
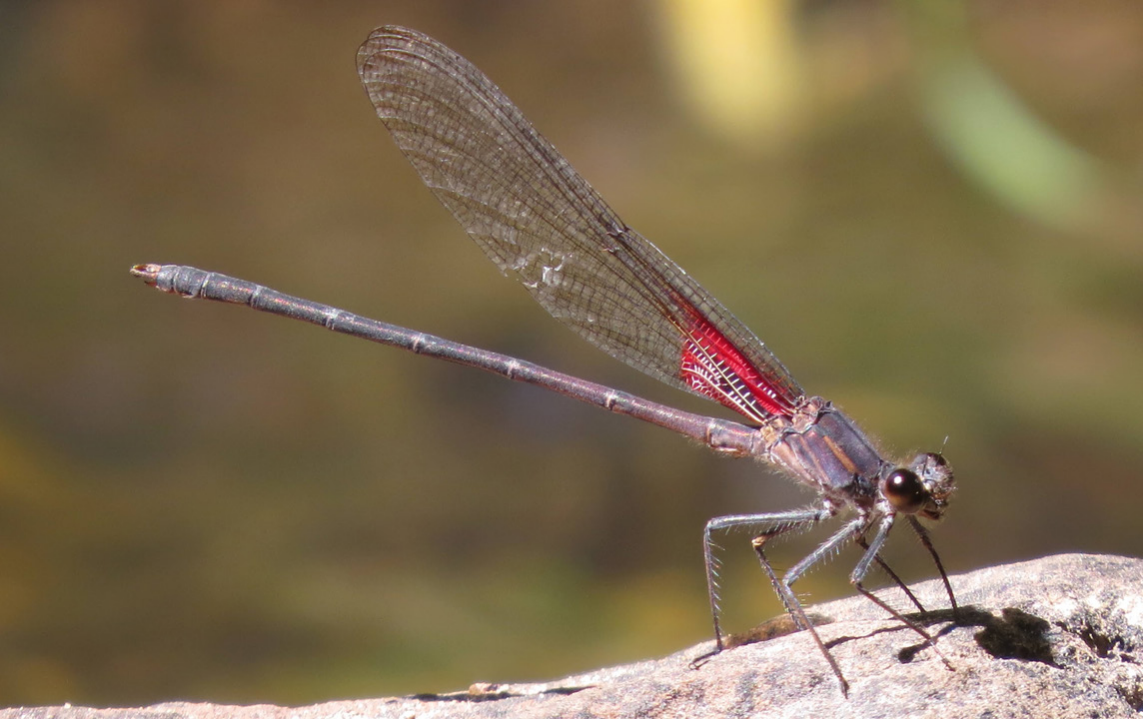
*Hetaerina vulnerata* individual (male) occupying a rocky, shaded stream in the Tonto National Forest area (photo credit: N.E.M.).

**FIGURE 2:**
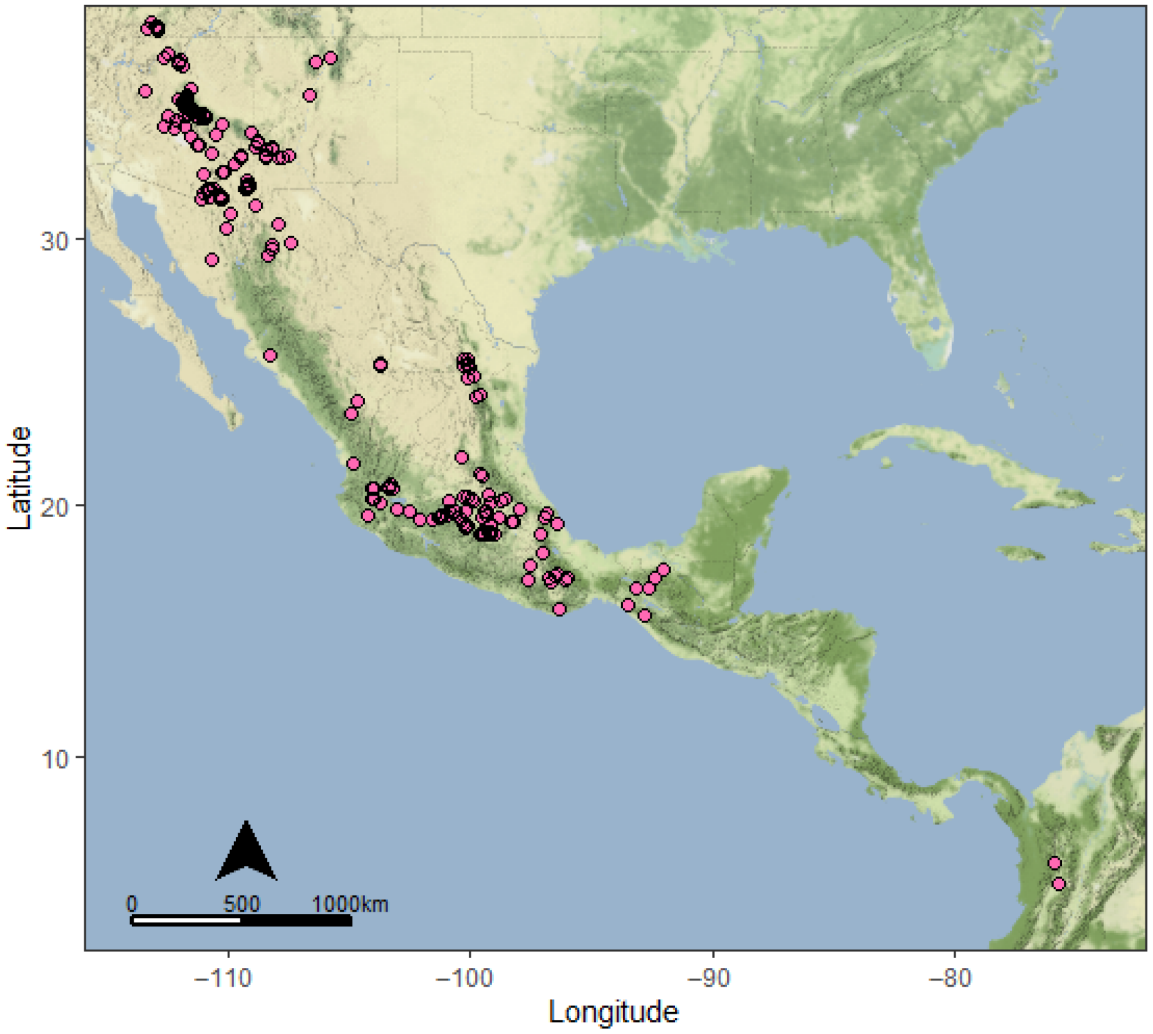
Range map for *Hetaerina vulnerata* from the southwestern United States to Colombia based on 516 Research Grade iNaturalist observations (pink points).

*Hetaerina vulnerata* Hagen in Selys, 1853 (Canyon Rubyspot damselfly; Figure 1) inhabits shaded streams in relatively high‐altitude areas (550–2000 m above sea level), ranging from Colombia up to the southwestern United States, where it is found in mountainous areas of Arizona, New Mexico, and Utah (Alcock, 1982; Johnson, 1973; Paulson, 2010; Stevens & Bailowitz, 2009) (Figure 2). It is considered a relatively slow‐flying damselfly with limited dispersal (Rivas et al., 2016). Spatial separation of stream drainages combined with the limited dispersal capacity of *H. vulnerata* likely induces population isolation and genetic differentiation, which could be exacerbated by climate change. Desiccation of streams would curtail gene flow in *H. vulnerata*, and SDMs can indicate such areas of sensitivity (Wang et al., 2008). Using paleoclimatic, current climatic, and projected future SDMs, we should be able to identify patterns in the current habitat for *H. vulnerata* that may indicate genetic differentiation or isolation related to geographic distance or availability of suitable environmental conditions.

We used locality records of *H. vulnerata* from vetted databases to build species distribution models under past, current, and projected future climates to quantify changes in potential niche space, identify possible climate refugia, and explain any current patterns of genetic differentiation. Because *H. vulnerata* are high‐altitude, stream‐specialist damselflies with populations in isolated, montane areas surrounded by lowland desert in the southwestern United States, we hypothesized that these populations would exhibit genetic differentiation shaped by shared climatic conditions. A separate model was created based on climate and scenopoetic predictors to predict the number of *H. vulnerata* subpopulations; genetic similarity/dissimilarity was expected to be reflected by separation between mountain ranges and a lack of aquatic habitat. Models constructed to examine this portion of the species' range should be robust because of the widespread data available for the United States (occurrence points and environmental data) relative to the rest of the species' distribution.

## 2 MATERIALS AND METHODS

### 2.1 Model species

*Hetaerina* (rubyspots) are the most speciose genus (39 spp.) of Calopterygidae in the New World (Garrison et al., 2010; Paulson et al., 2022) and is most diverse in South and Central America. *Hetaerina* are estimated to have diverged from other calopterygids ~36 million years ago, with *H. vulnerata* diverging ~10 million years ago (Standring et al., 2022). There are only four species of this genus in the United States: *H. calverti* Vega‐Sánchez, Mendoza‐Cuenca, & González‐Rodríguez, 2020 (Cryptic Rubyspot), *H. titia* (Drury, 1773) (Smoky Rubyspot), *H. americana* (Fabricius, 1798) (American Rubyspot), and *H. vulnerata* (Canyon Rubyspot). *Hetaerina calverti* was only recently described, being split from *H. americana*, so its full range is currently unclear (Vega‐Sánchez et al., 2020, 2023). Of the remaining three species, *H. americana* has the most widespread distribution in the United States whereas *H. vulnerata* has the most restricted range because of its habitat specificity (Garrison, 1990; Stevens & Bailowitz, 2009).

*Hetaerina* typically inhabit small streams and rivers, and certain species, such as *H. vulnerata*, preferentially occupy shaded streams and rivers, using shaded riparian areas for thermoregulation, as nighttime refugia, and for postcopulatory resting (Córdoba‐Aguilar & Rocha‐Ortega, 2019; Garcia‐Garcia et al., 2017); occupation of forest habitat is thought to be the ancestral state for members of this genus (Standring et al., 2022). Fine‐scale microhabitat preference makes *H. vulnerata* an emblematic species for modeling distribution and population isolation effects across spatiotemporal and genomic scales.

### 2.2 Species distribution models

#### 2.2.1 Bioclimatic SDMs

We constructed SDMs for *Hetaerina vulnerata* in the United States portion of its range, representing the northernmost (and presumably most recent) segment colonized for this otherwise Meso‐American genus (Standring et al., 2022). For *H. vulnerata* occurrence data, only verified species identification data from OdonataCentral (https://www.odonatacentral.org), the Global Biodiversity Information Facility (https://www.gbif.org), iNaturalist (https://www.inaturalist.org), and the Arizona Dragonflies (http://azdragonfly.org/) databases were used. Points from iNaturalist were omitted if they were not verified by the community or if the approximate location range was imprecise (>200 m). There was a total of 516 occurrence points across the entire range of *H. vulnerata*, and 210 of these points were within the study extent. Occurrence data were thinned to include only one point per cell/km^2^, using the *spThin* package (Aiello‐Lammens et al., 2015) in R (R Core Team, 2021), thereby minimizing overestimation by the SDM by reducing aggregated occurrences. SDMs were constructed using MaxEnt (Phillips et al., 2021), projecting distributions from the Last Glacial Maximum, the Mid‐Holocene, current (1970–2000), and future (2060–2080). Models were developed with presence data from spatial coordinates and a suite of environmental data layers (Phillips et al., 2006).

The 19 bioclimatic variables in the WorldClim‐Global Climate version 2 data repository (Fick & Hijmans, 2017) were retrieved for current conditions at a resolution of 30 arcsec. We created a training extent buffer of accessible area (M) for *H. vulnerata* by generating a buffer (200 km radius) around each point, estimating potential areas where *H. vulnerata* may have occurred. This buffer size also picks up variation in the raster datasets, since they were at a 1 km^2^ resolution for current, MH, and future conditions. Likewise, this buffer size also encapsulates variation for the LGM, which had a resolution of ~4 km^2^. We clipped and masked each variable to the buffer extent in ArcGIS Pro 3.1.0 (Esri, Redlands, CA, USA). Because bioclimatic variables are highly correlated, we used Pearson's correlation coefficient in the R package *virtualspecies* (Maynard et al., 2015) to select a set of independent variables (Pearson's correlation coefficient < 0.7) to model the species' bioclimatic niche (Soberón & Nakamura, 2009). From this process, five variables were selected as an independent suite from other bioclimatic variables: Bio2, Bio4, Bio12, Bio15, and Bio17 (see Appendix 1 for variable descriptions). Different mathematical transformations (e.g., regularization multipliers from 1 to 10) and features were applied independently or in conjunction to the models (e.g., linear, quadratic, hinge, etc.) to create response curves. The SDM was trained and tested with an independent subset of occurrences (70% [*n* = 88] and 30% [*n* = 37], respectively) as well as the full, filtered dataset of occurrence points. Under current conditions, 310 models were calibrated, evaluated, trained, and tested in *kuenm* (Cobos et al., 2019) and MaxEnt. The model features with the highest area under the curve (AUC), lowest omission rate (OR), and lowest Akaike's information criterion corrected for low sample size (AICc) were selected as the best candidate models to be projected to other climate scenarios. We used a thresholding function to omit areas of unsuitable habitat, based on locations with <10% probability of suitable habitat in the training dataset. When the final model was created for each scenario, 10 SDM outputs were generated; the average of these replicates was used for comparisons between climate regimes (i.e., the final model was based on an average of 10 replicate SDMs).

Maintaining predictor consistency, the same variables and model features were used for LGM, MH, and future projections to determine how suitable habitat for *H. vulnerata* has shifted or changed over time. To hindcast the SDMs, data from WorldClim 1.4 under the CCSM4 general circulation model (GCMs) were downloaded at the available resolutions of 2.5 arcmin (LGM) and 30 arcsec (MH). The current model features and regularization multipliers were projected to these scenarios, and a 10% omission thresholding was applied to the paleoclimatic models to predict how climatic shifts from the LGM to current times may have potentially affected genetic similarity or dissimilarity between and among populations.

To forecast the SDMs, data from WorldClim 1.4 under the IPSL‐CM5A‐LR GCMs and Shared Socioeconomic Pathways (SSPs) were downloaded under the Climate Model Intercomparison Project Phase 6 (CMIP6) at a resolution of 30 arcsec. The IPSL‐CM5A‐LR model represents projected conditions of aridity with increased temperature and lower precipitation rates. The SSPs selected (370 and 585) were from the high end of the range, bracketing probable climate futures for the southwestern United States (Miller et al., 2021). As with the current and paleoclimatic data, we predicted the future distribution of *H. vulnerata* with the five bioclimatic variables distilled from *virtualspecies*, and the same feature classes and regularization multipliers from model development and thresholding were applied to the future SDM. Future projections allow us to make inferences as to how climatic shifts may result in distribution expansion or contraction and probable outcomes (i.e., extirpation and/or persistence) for *H. vulnerata* across its US range.

To quantify differences in bioclimatic SDMs, we used the Raster Calculator tool in ArcGIS Pro 3.1.0 to subtract the current model from each projected model. The output allowed us to visualize gains and losses in habitat in comparison to current distributions across this spatial extent.

#### 2.2.2 Grinnellian SDMs

**FIGURE 3:**
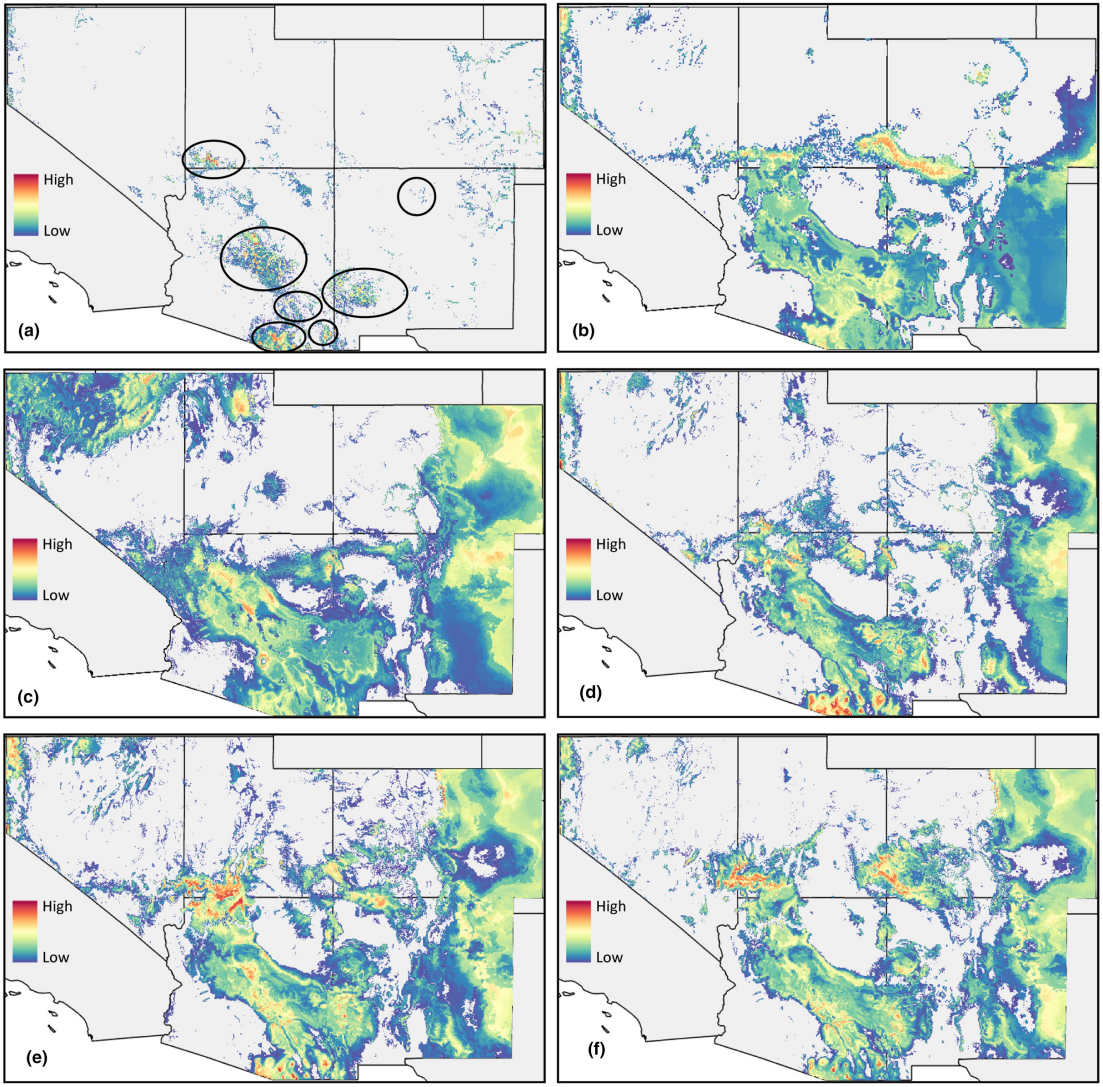
Grinnellian and Bioclimatic niche SDMs. Cool colors (e.g., blue) indicate low habitat suitability; warm colors (e.g., red/yellow) indicate high habitat suitability. A 10% threshold was applied to omit areas that are the most unsuitable for *Hetaerina vulnerata*. (a) Grinnellian niche with seven putative populations outlined in black ovals. (b) Last Glacial Maximum (GCM – CCSM‐ESM), (c) Mid‐Holocene (GCM – CCSM‐ESM), (d) Current climate, (e) Future climate (GCM – IPSL‐CM5A‐LR; SSP – 370), and (f) Future climate (GCM – IPSL‐CM5A‐LR; SSP – 585). Sampling locations and occurrence points are included in Figures 2 and 4.

To determine current putative population clustering across the landscape, we also built a Grinnellian niche (Grinnell, 1917; Soberón & Nakamura, 2009) species distribution model using 24 bioclimatic, physiographic (i.e., topography, streams, etc.), and habitat structure (tree canopy coverage) predictor variables. A Grinnellian niche is characterized by climate and necessary habitat present for a species to occur, opposed to strictly bioclimatic predictors. While habitat is predicated on climate, the variation in physiography, microhabitat, and other landscape features that are integral in a species' ecology provide more realistic constraints on a species' distribution. Resolution was at 30 arcsec for all layers. Elevation and flow direction data were retrieved from USGS HydroSHEDS (https://hydrosheds.cr.usgs.gov), and a slope layer was derived from the elevation data in ArcMap. Stream data for the southwestern United States were downloaded from the USGS National Hydrography Dataset (https://www.usgs.gov/national‐hydrography/access‐national‐hydrography‐products). Since adult *H. vulnerata* can disperse ~1 km away from streams (A.R.B. pers. obs.) and some occurrence data may be imprecise, a 2 km radius buffer layer (denoted as Stream) was generated from the stream polygons. Because Canyon Rubyspots occupy shaded streams, tree canopy coverage (TCC) data were retrieved and included here from the United States Forest Service (https://data.fs.usda.gov/geodata/rastergateway/treecanopycover/). Collectively, using both abiotic and habitat feature data provides a better likelihood of accurately modeling the species' current occurrence (Grinnellian niche). As before, a correlation analysis was conducted with *virtualspecies* to identify the most independent predictors (Bio2, Bio3, Bio5, Bio14, Bio15, Bio16, Direction, Slope, Stream, and TCC; Appendix 1). This SDM was used to identify current geographically discrete locations with putatively isolated populations (Figure 3a), which we then sampled for genetic analyses.

### 2.3 Sampling localities

**FIGURE 4:**
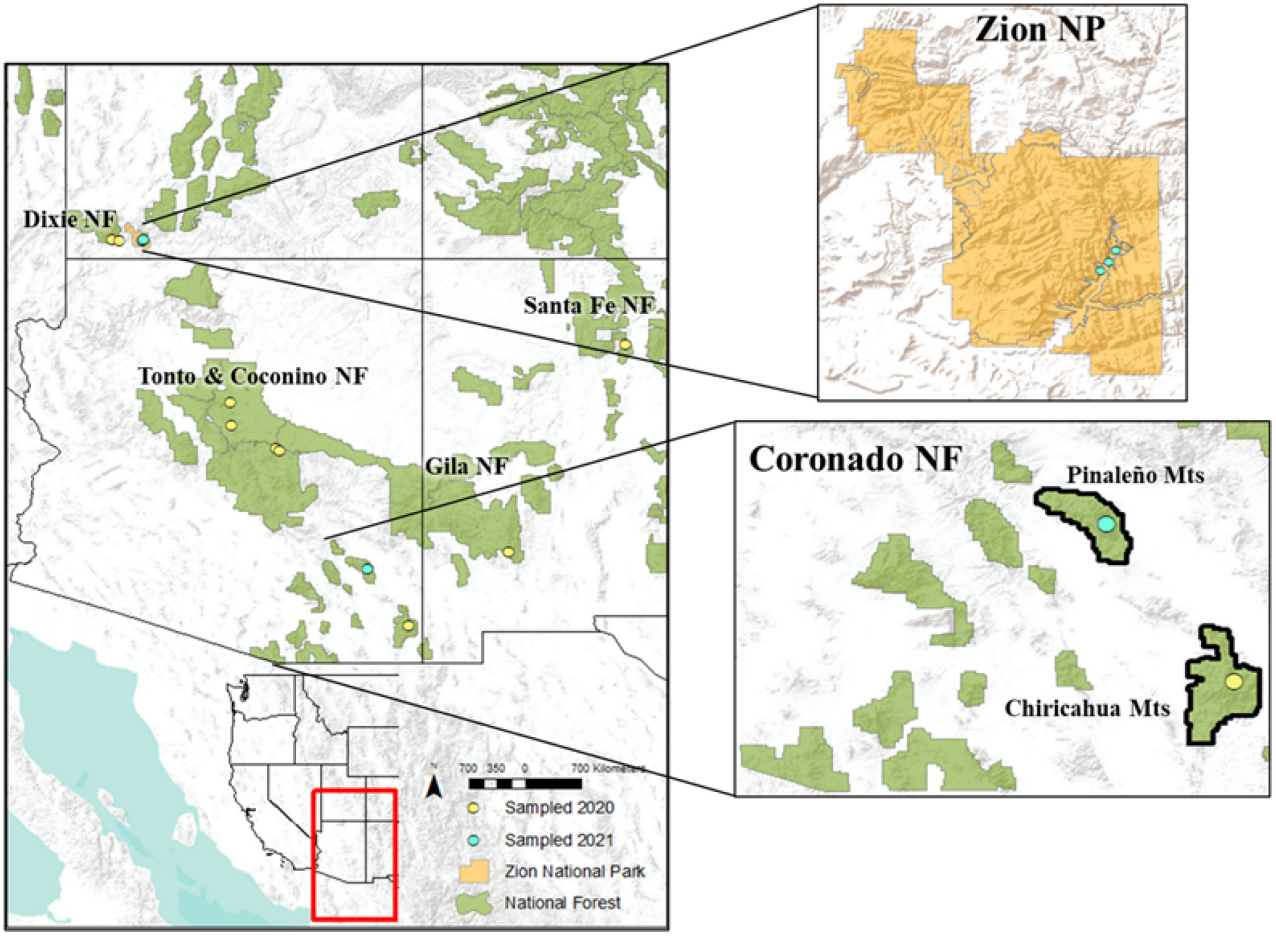
Sampling locations in the National Forests (NF) of the southwestern United States during the summers of 2020 (yellow points) and 2021 (light blue points). Inset maps are provided for Zion National Park (NP) and Coronado NF (black bounding box) to display locations that were proximal to each other and difficult to discern at the overall map extent.

The SDM output suggested the presence of seven *H. vulnerata* populations across the study extent, based on aggregation of occurrences and presence of unsuitable environmental conditions separating populations. From each of the seven putative clusters, at least one sampling location was established per cluster. Based on known occurrences from citizen‐science repositories within the putatively isolated populations identified by the SDM, sampling locations were chosen along shaded streams, a known habitat preference. Collecting was performed during times of high damselfly activity, that is, late morning to midday from July to September (Stevens & Bailowitz, 2009). We collected in the summers of 2020 (*n* = 101 individuals) and 2021 (*n* = 23) (Table 1, Figure 4). No damselflies were located in either year at one of the population clusters (the Santa Rita and Huachuca Mountains) because most streams in those mountain ranges were completely dry; from the remaining six clusters, we collected a minimum of 10 individuals (except for Dixie National Forest in Utah, where only *n* = 9 were able to be collected) to assess genomic structure via ddRAD‐Seq (Yadav et al., 2019). We collected adults rather than naiads/nymphs because of a lack of reliable taxonomic keys for identification, particularly for very early instars. The spatial distance between the putative population clusters ensured that even if an adult we collected did not emerge from the stream at which it was collected but instead had dispersed there from another stream (likely within the same watershed), it would still be representative of that population cluster. Adults were collected with aerial insect nets and immediately preserved in 100% ethanol. Within 2 h of collection, 100% ethanol was injected into the thorax of each individual for further DNA preservation (J.L.W. recommendation), and individuals were resubmerged in 100% ethanol.

**TABLE 1:** Putative populations visited by watershed and associated mountain range; coordinates are in latitude/longitude decimal degrees, and *n* corresponds to the number of individuals collected per watershed (total *n* = 124).

Population	Watershed	Mountain range	Elevation (m asl)	Coordinates (N, W)	*n*
Gila	Gallinas Creek	Black Mts.	2097	32.8977, −107.8244	16
Chiricahua	Cave Creek	Chiricahua Mts.	1792	31.8722, −109.2342	16
Pinaleño	Noon Creek	Pinaleño Mts.	1838	32.6511, −109.8127	13
Santa Fe	Pajarito Spring	Jemez Mts.	1700	35.8038, −106.1969	14
Tonto/Coconino	Horton Creek	Mogollon Rim	1713	34.3458, −111.09	17
	Christopher Creek	Mogollon Rim	1710	34.3088, −111.0383	10
	Wet Beaver Creek	Mogollon Rim	1161	34.6736, −111.7081	8
	Oak Creek	Mogollon Rim	1658	34.9958, −111.7378	11
Dixie/Zion	Leeds Canyon	Pine Valley Mts.	1414	37.2733, −113.3886	8
	Ash Creek	Pine Valley Mts.	1024	37.2569, −113.2861	1
	Virgin River	Colorado Plateau	1301	37.25, −112.95	10

From each locality sampled (*n* = 8), 10 individuals (nine from Dixie National Forest) were selected for DNA extraction and sequencing; these included two *H. americana* from Coconino National Forest and Dixie National Forest that were utilized as an outgroup for the species tree and for comparison with *F*_ST_ values to examine the degree of differentiation between *H. vulnerata* populations. Seventy‐eight *H. vulnerata* individuals were sequenced (Gila National Forest, NM = 10; Coronado National Forest [Chiricahua Mts. and Pinaleño Mts.], AZ = 20; Santa Fe National Forest, NM = 10; Tonto National Forest, AZ = 10; Coconino National Forest, AZ = 9; Utah [Dixie National Forest and Zion National Park] = 19).

### 2.4 DNA extraction and sequencing

DNA extraction occurred at Texas Tech University within 120 days of field collection. Individuals were retrieved from ethanol; once completely air dried, whole genomic DNA was extracted from thoracic muscle (Watts et al., 2007) using the Qiagen (Germantown, MD, USA) DNeasy Blood and Tissues kit, abiding by the protocol of the manufacturer for insect tissue. DNA concentration was quantified with an Invitrogen Qubit fluorometer. Whole genome extracts were outsourced to Admera Health, LLC (South Plainfield, NJ, USA) for double‐digest restriction‐site associated DNA sequencing (ddRAD‐seq; Peterson et al., 2012) library preparation with two restriction enzymes (NlaIII‐MluCI) and subsequent sequencing via Illumina Hi‐seq X with 150 bp for each paired end read.

### 2.5 Data analyses

#### 2.5.1 ddRAD‐seq data filtering

Demultiplexed raw sequences were filtered and aligned using the software Stacks v2.53 with default settings and a phred33 scoring (Catchen et al., 2013). Default parameters were used in Stacks, aside from some slight modifications of settings in ustacks and cstacks. The parameter for maximum distance permitted between nucleotides between each stack, implemented in ustacks, was adjusted to 4 (default = 2), and the same value was retained in cstacks for number of mismatched loci allowed between sample loci while the catalog was being constructed, as recommended by Catchen et al. (2013). In the populations program, the minimum percentage of individuals within (‐*r*) and across (‐*R*) populations required to process a locus for that population was 50%, and each locus had to be present in at least three populations (Cerca et al., 2021). The ‐‐write‐single‐snp flag was used to remove potentially linked single nucleotide polymorphisms (SNPs), and Stacks output files (e.g., VCF, Genepop, PHYLIP, etc.) were used for downstream analyses. Due to the amount of nonoverlapping RAD loci for the Pinaleño and Zion populations with other populations, we created three datasets of filtered SNPs: a complete set of all *Hetaerina* individuals (for *F*_ST_ and dendrogram to visualize whether individuals and populations cluster logically), a dataset containing *H. americana* but without the Pinaleño and Zion populations (for phylogeographic estimates), and a set lacking those two populations and lacking *H. americana* (for PCA and landscape‐scale metrics). Thus, analyses such as PCA, IQ‐TREE, and SNAPP (phylogeographic analysis) that are sensitive to the absence of loci (and, therefore, missing alleles for that locus) would not erroneously cluster the Zion and Pinaleño populations together, since they showed relatively high *F*_ST_ values between each other.

To determine the certainty of our filtering from Stacks, we implemented SNPfiltR (DeRaad, 2022). Using our filtered SNP dataset for the *H. vulnerata* individuals, except the Zion and Pinaleño populations, we removed alleles that had a genotype quality <30 and less than a minimum depth of 5, with the “hard_filter” function. Minor alleles ≤4 copies were removed with the “min_mac” function. Heterozygous genotypes that were outside the 0.25–0.75 range per individual were converted to NA values using the “filter_allele_balance” function. Genotypes/reads with over 90% missingness and SNPs with over 50% missingness were removed. The VCF output was used for some downstream population genetic analyses (i.e., PCA and Structure) to corroborate outputs from the dataset generated with Stacks.

#### 2.5.2 Population genetic analyses

Since individuals collected from a few of the putative populations were from the same stream or watershed, we used the *related* (Pew et al., 2015) package in R to identify any potentially related individuals. The *related* package determines combinations of coancestry and whether individuals are parent–offspring, siblings, or cousins via relatedness estimates from Li et al. (1993) and Wang (2002). Pairs determined as parent–offspring (coancestry > 0.5) would be eliminated from other analyses. Because of the recognized stable‐edge hypothesis, we also calculated expected and observed heterozygosity (*H*_E_ and *H*_O_, respectively) to determine probable patterns of dispersal and population settlement for *H. vulnerata* across this portion of its range, using the *hierfstat* (Goudet, 2021) package. To calculate the average number of segregating sites per population, we used the π estimate, which was quantified in the populations program of Stacks; with π, there are no underlying assumptions about population structure.

To estimate hierarchical clustering of our sequenced individuals, we used the hclust analysis with a “ward.D2” method and Euclidean distance in the *stats* package to create a dendrogram with the dataset containing all *Hetaerina* individuals. Then, the as.phylo function in the *ape* package (Paradis & Schliep, 2019) was used to visualize the dendrogram akin to a phylogenetic tree. *F*_ST_ is a metric to indicate degree of genetic differentiation among populations and is scaled from 0 (panmixia) to 1 (complete differentiation). We used the *hierfstat* package with Nei's (1987) pairwise genetic distance to calculate *F*_ST_. We also calculated *F*_ST_ with all *H. vulnerata* individuals and the *H. americana* samples; in doing so, we could infer estimates of potential speciation trajectories, especially for the more isolated populations. To visualize genetic patterns in the dataset lacking Pinaleño and Zion individuals, we used a principal component analysis (PCA) to estimate clustering of populations. In addition, we used *Structure* v.2.3.4 (Pritchard et al., 2000) to estimate structuring of populations (with a burn‐in of 5000, 100,000 MCMC replications, and *K*‐values from 1 to 7 across 10 repetitions), and *Distruct* v.1.1 (Rosenberg, 2004) was used to visualize and customize outputs from *Structure*. *structureHarvester* v0.7 (Earl & vonHoldt, 2012) was used to concatenate the outputs from each *K* and repetition per *K*, running summary statistics and the Evanno delta K method (Evanno et al., 2005) to determine the best *K*‐value. However, the *Structure* runs with *K* = 2–4 were plotted.

#### 2.5.3 Landscape genomic analyses

Isolation by distance (IBD; based on geographical distances in decimal degrees and Nei's (1987) pairwise genetic distance) was determined via a Mantel test in R with the *vegan* package (Oksanen et al., 2013); a Pearson correlation method and 999 permutations were used. Isolation by environment (IBE) was modeled with a maximum likelihood population effects (MLPE) model; each model was run as a function of log‐transformed Nei's (1987) pairwise genetic distance (to achieve normality) with environmental variables as the predictor and population as a random effect. The models were evaluated with AIC and BIC. The best model was selected and hypothesized to contribute toward IBE for *H. vulnerata*. To generate the intervening landscape environmental predictors, we plotted geographic coordinates, created links between each point based on Euclidean distance, used a 2 km radius buffer to account for potential dispersal, and extracted mean values from within the buffer for each raster dataset. Two niche models were tested: bioclimatic niche and Grinnellian niche. The initial variables for the niche models were the same as the SDM variables, but we used a variance inflation factor (VIF) to further remove correlated environmental variables, since correlations might be different due to a finer scale than the SDM.

#### 2.5.4 Phylogeographic analyses

Because we were inferring intraspecific phylogeographic patterns for *H. vulnerata* based on the SDM output, we also constructed a Bayesian phylogeny in BEAST v2.7.5 (Bouckaert, 2022; Bouckaert et al., 2019; Bryant et al., 2012), with two *H. americana* individuals as the outgroup. We used the package SNAPP v1.6.1 (SNP and AFLP Package for Phylogenetic analysis) to build and visualize a coalescent species tree for sampled *H. vulnerata* populations. Individuals were lumped into geographic clades to minimize computational demand. We used the same number of SNPs (*n* = 9240) as from the dataset lacking Zion and Pinaleño individuals. We ran the model with a gamma rate prior, *α* = 2, *β* = 200 (Leaché et al., 2014) and a MCMC chain length of 5,000,000, sampling every 1000 trees (Bryson et al., 2016). MCMC convergence verification was performed with Tracer to ensure that the effective sample size (ESS) values for all parameters were > 300 for each independent run; the high chain length values were to achieve adequate ESS scores. LogCombiner was used to combine all the log and tree files to analyze collective log files in Tracer (ESS > 1000) and to determine the best consensus tree from multiple runs from the consolidated tree files. We used TreeAnnotator to calculate the posterior distribution of *H. vulnerata* clades with a maximum credibility tree and 10% burn‐in.

To corroborate our Bayesian phylogeny, we used a maximum likelihood method, IQ‐TREE v2.2.2.6 (Minh et al., 2020). Consensus sequences for each population were concatenated, retaining variant and non‐variant sites. We used IQ‐TREE for mutation model selection (Kalyaanamoorthy et al., 2017), and subsequently, we performed an ultrafast bootstrap approximation (Hoang et al., 2018) with 10,000 bootstrap replicates (Vega‐Sánchez et al., 2023). The consensus tree contained bootstrap likelihood estimates of branch support. The consensus tree outputs from SNAPP and IQ‐TREE were visualized with FigTree v.1.4.4 (http://tree.bio.ed.ac.uk/software/figtree/).

## 3 RESULTS

### 3.1 Species distribution models

#### 3.1.1 Bioclimatic SDMs

Based on model fit and complexity estimates of feature classes used (see Appendix 2 for AICc, OR, and AUC details for candidate SDMs constructed with *kuenm*) during calibration, the best current model had a threshold feature applied and a regularization multiplier of 1, and this feature was used for all projection scenarios. Permutation importance for the variables included in the paleoclimate, current, and future SDMs can be found in Appendix 3.

During the LGM and MH, there was a contiguous distribution of suitable habitat throughout most of Arizona and New Mexico; disjunct but suitable habitat was also found in Utah, Colorado, and Nevada (Figure 3b,c). In current and future models, the amount of suitable habitat (based on climate) is waning (Figure 3d–f) in comparison to the LGM and MH models, with latitudinal shifts in future habitat. Currently, there is suitable habitat between central Arizona/New Mexico populations and the southern Utah population. Habitat suitability increased from the LGM to current and future conditions in the Lincoln National Forest area (New Mexico), indicating plausible climate refugia there. Suitable habitat in and around Santa Fe National Forest was not reliably present in any of the climate models. Interestingly, eastern New Mexico and Colorado display suitable bioclimatic conditions, but this area is classified as the Great Plains, which is a prairie ecosystem with very little topographic variation.

Based on the current SDM, Lincoln National Forest (New Mexico) and Pine Valley in Dixie National Forest (Utah) were indicated as areas of current high habitat suitability; yet, presence of *H. vulnerata* in those areas has not been documented. We visited these localities in 2021 to determine species presence but did not detect any *H. vulnerata* (possibly due to limited sampling time, inability of *H. vulnerata* to successfully disperse to this area, recent changes in habitat suitability due to forest fires, or over‐estimation predicted by the model). Santa Fe National Forest (New Mexico) does not show high climatic suitability; yet individuals were present when we sampled this area.

Future climate projections from both SSP scenarios for *H. vulnerata* display a dwindling amount of suitable habitat between some populations (i.e., the Madrean Archipelago of southern Arizona), with climatic niche conditions putatively shifting up in latitude. The central portion of Arizona and New Mexico shows contiguity of habitat, following a trend seen in paleoclimate and current SDMs. Populations in New Mexico (disregarding the Gila National Forest area) may be extirpated from a loss of suitable climate, although they have persisted with minimal climatic suitability. Interestingly, the potential climate refugia (Lincoln National Forest area) remains intact, but with minimal climatic connectivity. Across the models, there was consistent loss in suitable habitat relative to current SDMs in the Madrean Archipelago region of southern Arizona for *H. vulnerata*, and gains in suitable habitat were variable across the northern portion of their range (Appendix 4).

#### 3.1.2 Grinnellian SDM

Based on model fit and complexity estimates of feature classes used (see Appendix 2 for AICc, OR, and AUC details for candidate SDMs constructed with *kuenm*) during calibration, the best current model had a product and threshold feature applied and a regularization multiplier of 1. Permutation importance for the variables included in the Grinnellian SDMs can be found in Appendix 5.

The Grinnellian niche SDM was used to indicate putative population clusters. Based on aggregated suitable habitat (via scenopoetic variables), we inferred that these were subpopulations of *H. vulnerata* (Figure 3a) from which we sampled. Suitable habitat is disjunct between populations in central Arizona and New Mexico. Mountain ranges in southern Arizona have intermittent suitable habitat between populations. The northern New Mexico and southern Utah populations are separated by unsuitable habitat from the rest of the range of *H. vulnerata*; these northernmost populations are conceivably the most isolated. Indeed, suitable habitat in northern New Mexico is very sparse and disjunct. Contradicting the climatic SDMs, there is negligible suitable habitat in the Lincoln National Forest area, suggesting that habitat conditions are not met for *H. vulnerata* to be present there. Only the bioclimatic niche SDMs indicate the Lincoln National Forest as habitat refugia.

### 3.2 Genomic analyses

#### 3.2.1 ddRAD‐seq filtering results

We recovered 6.16–36.5 Mbp raw sequences across all samples, and based on a quality scoring of phred33 for process_radtags filtering of fastq files, 99.4% of sequences were retained (6.15–36.2 Mbp). Following Stacks processing and parameter adjustment, we retained 9971 loci and 9240 SNPs determined to be unique across *H. vulnerata* and *H. americana* individuals (Zion and Pinaleño populations excluded). Including the Zion and Pinaleño populations, we retained 2080 loci and 1787 SNPs. Excluding *H. americana*, Zion, and Pinaleño populations, there were 10,940 loci and 10,075 SNPs. These SNP datasets (Appendix 6) were then used to estimate clustering, *F*_ST_, and population isolation (see Methods for how they were implemented with different analyses). The average coverage was 7.46×, as reported from ustacks (Table 2). Further filtering with SNPfiltR after Stacks resulted in 2940 variants in the *H. vulnerata* dataset excluding *H. americana* and the Zion and Pinaleño populations (see Appendix 7 for percent missing data from both datasets). When we applied this filtering to the SNP dataset with *H. americana*, they did not pass the filtering requirements. Since these individuals are necessary for the phylogeographic analyses, we used the SNP data generated from the Stacks pipeline.

**TABLE 2:** Mean depth coverage of all loci with variable sites per putative population, as reported by the ustacks program.

	Mean depth coverage	*n*
Gila	7.93×	10
Chiricahua	8.03×	10
Pinaleño	11.5×	10
Santa Fe	7.70×	10
Tonto/Coconino	7.66×	19
Dixie/Zion	9.39×	19

*Note*: The number of individuals sequenced per locality is indicated by *n*.

#### 3.2.2 Population statistics

As determined via relatedness analyses, the individuals we collected within populations were not parent–offspring or siblings (all coancestry values < 0.5). *H*_E_ and *H*_O_ showed a latitudinal cline, being highest in the southernmost locations (Table 3). The northernmost populations (Santa Fe and Utah) had the lowest estimates of π and have thus accrued fewer sequence changes compared to other populations of *H. vulnerata*. The dendrogram generated with a “wald.D2” method and Euclidean distance indicates that each population clusters together accordingly, with one Gila individual appearing in the Tonto cluster. Zion and Pinaleño populations erroneously cluster together, nearest to the *H. americana* outgroup (Appendix 8).

**TABLE 3:** Mean number of segregating sites (*π*), observed heterozygosity (*H*_O_), and expected heterozygosity (*H*_E_) values per locality.

	*H*_O_	*H*_E_	*π*	Latitude
Utah	0.066	0.068	0.074	37.2
Santa Fe	0.067	0.075	0.080	35.8
Coconino	0.094	0.106	0.116	34.6
Tonto	0.096	0.109	0.119	34.3
Gila	0.103	0.116	0.125	32.8
Chiricahua	0.122	0.148	0.161	31.8

*Note*: Latitude is included to demonstrate the association between heterozygosity estimates and approximate latitudinal distribution of *Hetaerina vulnerata*, ordered by descending latitude values.

**FIGURE 5:**
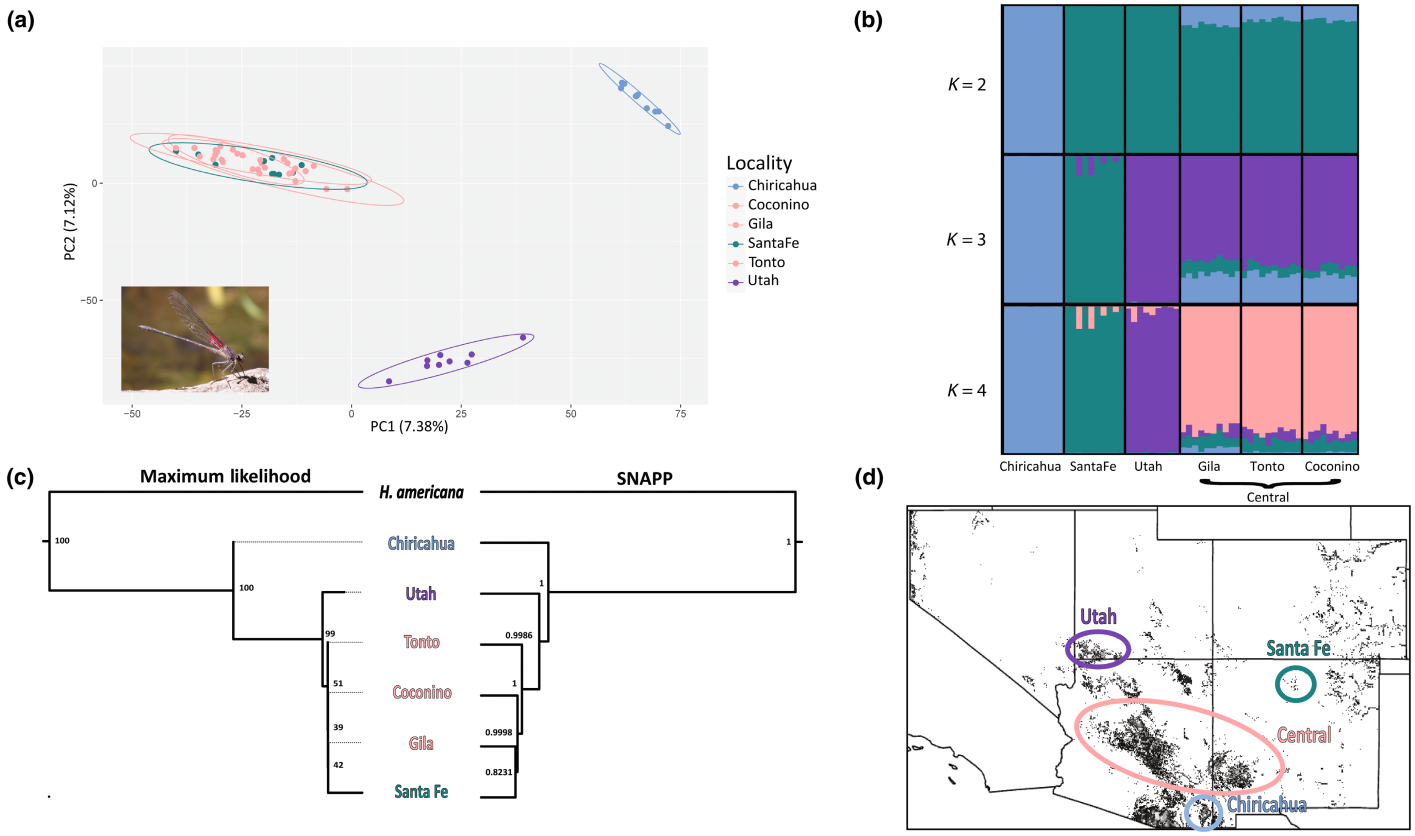
Grinnellian SDM predictions for *Hetaerina vulnerata* are partially corroborated by genomic data. (a) PCA of individuals, with confidence ellipsoids. Populations do overlap, but they are discernible by differences in color (peach and sea green) (b) *Structure* plots of *K* = 2–4, showing that four subpopulations parse out at *K* = 4. (c) Maximum likelihood and SNAPP phylogeographic trees with strong support for most branches. (d) Grinnellian SDM (from Figure 3) in grayscale with labeled populations and ovals representing putative subpopulations, consistent with the *Structure* output. Color scheme is coordinated with *K* = 4 *Structure* plot.

*F*_ST_ values were low (<0.05) between proximal populations, and differentiation increased with geographic distance or with separation from unsuitable habitat (Table 4). *F*_ST_ for *H. americana* compared to *H. vulnerata* was >0.8 (Appendix 9), indicating a very high value as expected between species. The Chiricahua Mts. and Santa Fe National Forest clusters had markedly consistent genetic differentiation between putative clusters. *F*_ST_ values indicate probable gene flow across the Gila National Forest, Tonto National Forest, Coconino National Forest, and Pinaleño Mountains. Furthermore, the genetic differentiation of the putative populations from Utah demonstrates similarity by proximity with the central Arizona populations (Appendix 9). Thus, based on *F*_ST_ values, there are conceivably four population clusters. More conservatively, the PCA suggested that there are three population clusters (Figure 5a); the Santa Fe populations lumped with the Gila, Tonto, and Coconino populations, with Chiricahua and Utah individuals representing their own cluster. PC1 and PC2 explained 7.38% and 7.12% of model variance, respectively. Outputs from *Structure* suggested that *K* = 4 based on the lowest natural‐log likelihood from the *K* = 1–7 iterations; four populations parsed out (Figure 5b) when plotting *K* = 2–4. Chiricahua, Santa Fe, and Utah represented their own populations from *K* = 2–4, consistent with *F*_ST_ values. PCA and Structure results from the SNPfiltR dataset closely resemble these results, with the outputs showing four populations (Appendix 10).

**TABLE 4:** Mean *F*_ST_ values per locality sampled, using Nei's (1987) pairwise distance formula on the bottom portion of the matrix.

	Gila	Chiricahua	Santa Fe	Tonto	Coconino	Utah
Gila	—	1.743	3.331	3.572	4.440	6.724
Chiricahua	0.232	—	4.969	3.092	4.003	6.537
Santa Fe	0.194	0.354	—	5.106	5.599	6.906
Tonto	0.009	0.243	0.201	—	0.918	3.449
Coconino	0.012	0.237	0.202	0.003	—	2.559
Utah	0.177	0.357	0.348	0.175	0.175	—

*Note*: The top part of the matrix is Euclidean distance in decimal degrees between populations.

#### 3.2.3 Isolation models

Isolation by distance quantifies whether geographically proximate populations are more closely related than those separated by larger distances, regardless of landscape factors, whereas IBE takes landscape and environmental factors into account for impacting genetic differentiation. A Mantel test indicated that isolation by distance was marginally significant (*p* = .0667, *r* = 0.513). IBE models via the MLPE analysis indicated that the Grinnellian niche was the best model, based on the AIC and BIC scores (Appendix 11). Tree canopy coverage was the only variable with confidence intervals that did not overlap zero (Appendices 12 and 13), meaning that tree canopy coverage significantly negatively influenced gene flow between populations. This is unsurprising, as the nearby populations had lower *F*_ST_ values than did distal populations, and tree canopy coverage was higher between nearby populations (e.g., Gila, Tonto, and Coconino National Forests are all near each other and have abundant tree canopy coverage; Appendix 14). Variance in the bioclimatic predictors may also have been weak due to the fine spatial extent and 2 km radius buffer (each raster was 1 km in dimension).

#### 3.2.4 Phylogeographic structuring

Posterior probabilities and maximum likelihood values indicated good support for population assignment (Figure 5c). However, the posterior probabilities were 0.82 between the Gila and Santa Fe populations, so the support for that clade is low. Similarly, with a mutation model of HKY + F + I, the bootstrap supports were 100 for phylogenetic assignments of *H. americana*, Chiricahua, and Utah populations; there was very weak support for the branches containing Tonto, Coconino, Santa Fe, and Gila populations (Figure 5c). The outgroup was resolved as monophyletic for both phylogenies.

## 4 DISCUSSION

*Hetaerina vulnerata* is a charismatic species that is restricted to high‐elevation streams in the desert southwestern United States of its northernmost range. We aimed to predict genetic differentiation using bioclimatic and Grinnellian SDMs to understand their past and current niche conditions, and how this will shift in the future. With SNP data, we uncovered that SDMs provide a useful, a priori framework for detecting areas of genetic differentiation, and found that heterogeneity of habitat between populations is driving current differentiation (Figure 5). This was supported by degrees of genetic differentiation and phylogeographic patterns that reflected distribution patterns found in our SDMs (Figure 5d). The SDMs presented here are currently the only models explicitly generated for an odonate in this region of the southwestern United States that consider past, current, and future climate regimes (but see Abbott et al., 2022 for a continental‐scale SDM overview), although a recent study did explore niche models for current distributions of the *H. americana* complex in Central and North America (Vega‐Sánchez et al., 2023). Indeed, our study is the first to use *H. vulnerata* as a model taxon to examine effects of paleoclimate, habitat isolation, and climate change, and how these factors are impacting the populations at the genetic level.

### 4.1 Bioclimatic SDMs versus Grinnellian SDMs

From the Last Glacial Maximum to the Mid‐Holocene, there was likely potential for gene flow among populations in central Arizona, New Mexico, and southern Utah, based on the presence of suitable habitat, but it is fragmented based on the current SDM. During the LGM, previous models and estimations indicated that conditions in the southwestern United States experienced a higher net increase of precipitation (Lora et al., 2017) and thus continued wet conditions that would have supported aquatic environments. During the MH, more arid conditions dominated the climatic landscape across the western United States (Steponaitis et al., 2015), but these estimations do not seem to have impacted the climatic distribution of *H. vulnerata*. Regardless, historical conditions provide a robust explanation for the highly contiguous suitable climatic niche during the LGM, which began to wane from the MH to now. Xue et al. (2017) documented a similar trend of habitat suitability increasing around the LGM and MH for an island endemic damselfly, with a more recent habitat reduction. Patterns of suitability were further confirmed with mitochondrial DNA data and genetic diversity metrics (Xue et al., 2017).

*Hetaerina vulnerata* populations in southern Utah have experienced isolation based on a suitable habitat reduction from the LGM to MH (Figure 3b,c), and *F*_ST_ are values consistent with this climatic trend and probable isolation from central Arizona and New Mexico (Table 4). Unstable suitable habitat for the Santa Fe population was inferred from the SDM, reflected in genetic data that demonstrated high differentiation by geographic distance. In addition, the paleoclimate models contributed hypotheses about how the genetic data will appear based on contiguity of suitable habitat or lack thereof.

Our future projected SDMs displayed moderately exacerbated arid conditions in the southwestern United States (Miller et al., 2021; Yanahan & Moore, 2019), specifically in the Madrean Archipelago region (Figure 3e,f), which is naturally more isolated for populations due to these mountains punctuating an otherwise flat, xeric landscape (Abernethy et al., 2022; Manthey & Moyle, 2015; Phillipsen et al., 2015). Populations in the Santa Fe National Forest area are not anticipated to gain suitable habitat, which may lead to their extirpation or uncertainty in population persistence (Betts et al., 2014; Bosso et al., 2018; Travis, 2003; Urbani et al., 2017). Future habitat suitability for *H. vulnerata* will likely be found at higher altitudes than presently, if shady streams are available, and future models display an elevational increase in suitable habitat.

Elucidation of how high‐elevation, aquatic insect taxa may react to broad‐scale (both spatial and temporal) climate change is a challenge (see review by Birrell et al., 2020). Nevertheless, high‐altitude specialists seldom disperse latitudinally from mountains (Leach et al., 2015; Siefert et al., 2015), supporting the notion that colonization of higher elevations is more probable (Chen et al., 2011; Freeman et al., 2018). SDMs published by Collins and McIntyre (2017) and Bellis et al. (2021) underscore the effects of climate change for odonates, suggesting loss of suitable habitat, and the results presented here point toward some diminishing suitable habitat for *H. vulnerata* with constriction by elevation. It is worth noting that there are several climatic models available for future conditions, and each might yield different outcomes (Miller et al., 2021; Yanahan & Moore, 2019). Furthermore, the amount of accessible area has a direct influence on how much suitable habitat is present in SDMs (Barve et al., 2011), but 200 km is likely an appropriate amount for *H. vulnerata*. In addition, the Grinnellian niche SDM presents an ominous outcome for *H. vulnerata* if habitat predictors are not present in a commensurate density to support *H. vulnerata* populations under future conditions.

### 4.2 Genetic results integrated with SDMs

Isolation by environment is caused by the unavailability of suitable habitat between isolated populations, which curtails migration and gene flow between populations (Wang & Bradburd, 2014). Since *H. vulnerata*, like most lotic specialists, disperse along watersheds, the ephemeral streams of the southwestern United States could be an environmental factor isolating populations (Grewe et al., 2012; Malison et al., 2022), although it was not captured as such in the MLPE models that used Euclidean distance between populations. Previous studies in the southwestern United States have posited that low‐dispersing aquatic insects would show high isolation and genetic differentiation from a lack of gene flow (Abernethy et al., 2022; Phillipsen et al., 2015). In addition, mild temperatures during the autumn and winter in the southwestern United States is associated with the presence of some *H. vulnerata* at low altitudes (vetted observations on iNaturalist), but it is not yet known if these individuals are dispersing to other subpopulations because of a possible lack of structural connectivity between watersheds. It is worth noting that more extensive sampling for *H. vulnerata* at different independent localities at the current spatial extent and the entirety of their distribution could show more signs of IBD because a finer sampling resolution could elucidate more accurate population genetic estimates.

The putative population clusters identified by the Grinnellian SDM were partially corroborated via *F*_ST_ and *Structure* (*k* = 4), as well as the PCA, SNAPP, and IQ‐TREE (*k* = 3). Populations separated by distance and xeric habitat displayed genetic differences. Because the Grinnellian SDM relies on scenopoetic variables, it produced a reliable model to predict genetic results for *H. vulnerata*. In combination, more realistic models (i.e., ones built with more than just bioclimatic variables) and genetic data can uncover population arrangement across the landscape. Comparing our two niche modeling approaches, the Grinnellian SDM indicated a paucity of suitable habitat between populations, which is more characteristic of this landscape and explains the potential lack of gene flow: low elevations might meet climatic conditions but lack tree coverage and aquatic habitat necessary for *H. vulnerata* populations to persist. With more fine‐scale thematic data, SDMs will be further refined to address questions at smaller scales, which will aid in current models and estimating how habitat will change in the future.

The populations in the Chiricahua Mountains, Santa Fe National Forest, and southern Utah exhibited differences from other populations. The *H. vulnerata* population in the Chiricahua Mountains had markedly high *F*_ST_ values compared to other populations, suggesting a speciation trajectory based on *H. americana F*_ST_ values. The *F*_ST_ values for Chiricahua‐Utah and Chiricahua‐Santa Fe pairs were similar to those reported by Vega‐Sánchez et al. (2023), where members of the *H. americana* species complex had a high degree of differentiation between the North and South populations (*F*_ST_ > 0.3). Regarding *H. vulnerata*, future SDMs show exacerbated isolation for the southern Arizona population (Figure 3e,f), which might lead to allopatric speciation. Since *F*_ST_ was low (<0.05) between the Pinaleño Mts, Gila, Coconino, and Tonto National Forest areas (Appendix 9), it is conceivable that there is contemporary or historically recent gene flow and these localities represent one population; the nearby Pinaleño subpopulation was likely recently isolated from populations occupying the Mogollon Rim (Hill & Unckless, 2021; Manthey & Moyle, 2015) and the Chiricahua population (Appendix 9). However, odonates have been shown to exhibit slow evolutionary rates (Kjer et al., 2006), which may not reflect the overall length of time since isolation, but evolutionary rates of the genome depend on the genomic feature that is under evolutionary pressures. Regardless, the Chiricahua Mts., Santa Fe, and southern Utah populations were the most isolated at the genetic level, and future climatic isolation and loss of habitat should exacerbate this differentiation (Figure 3e,f). The presence of such differentiation between putative subpopulations suggests that although nearby subpopulations share some similarities, a lack of gene flow due to extrinsic landscape factors has potentially resulted in clustering (Abernethy et al., 2022; Medina et al., 2021; Phillipsen et al., 2015).

The southernmost population (Chiricahua Mts.) had the highest heterozygosity values (Table 3). It is presumed that this area was colonized first in the southwestern United States, genetically diversifying longer. However, during the LGM, *H. vulnerata* had contiguous climatic suitability throughout most of its range, which is consistent with genetic similarity of central populations, and during the MH, we see a resemblance like that of current distributions. The Utah population had highly unsuitable habitat, and this was reflected in the genetic differentiation of this population with others. In addition, heterozygosity demonstrated a latitudinal cline along a north–south gradient (Table 3) consistent with northward colonization and a stepping‐stone model of dispersal (Kimura & Weiss, 1964). Future projections indicate that the southern Arizona populations are likely to become more isolated, which may result in an increase of homozygosity and would be potentially deleterious for population persistence for *H. vulnerata* across its current North American range.

Although some organisms are expected to shift their range in response to climate change (Lyons et al., 2019), this is species‐specific (Angert et al., 2011; Betts et al., 2014; Cacciapaglia & van Woesik, 2018). Range shifts compounded with climate change are anticipated to negatively affect aquatic organisms at higher elevations (Bellis et al., 2021; Slavich et al., 2014; Timoner et al., 2021), such as *H. vulnerata*. Moreover, even if suitable higher elevation areas are present, habitat selection at a fine scale (based on whether a localized site provides appropriate resources) will dictate whether a population is able to persist there (Hillman et al., 2014; Kirkton & Schultz, 2001; Polic et al., 2014). However, climatic SDMs typically cannot capture such microhabitat details (Collins & McIntyre, 2015), which might explain the scarcity of suitable habitat in the northern New Mexico population through time and the discordance between the Grinnellian and bioclimatic SDMs.

*Hetaerina vulnerata* is restricted and isolated by tree canopy coverage (Appendices 6, 7, 8). Males use shaded streams for territoriality and thermoregulation; after copulation, females of this genus are placed underwater by the male and they oviposit in submerged tree root mats (Alcock, 1982; Biddy et al., 2020; Eberhard, 1986; Johnson, 1961). The presence of riparian trees is integral in their life cycle, and the inclusion of this biologically meaningful variable in the Grinnellian SDM strengthens its predictive power. (However, it is worth noting that at 30 arcsec resolution, riparian trees cannot be distinguished from other forest types.) Under anticipated climate changes in the southwestern United States, wildfires are expected to increase, imperiling tree populations (Friggens & Finch, 2015). For consideration, all the bioclimatic niche SDMs did not include habitat structure variables, and current climate variables represent 1970–2000 conditions; thus, the paleoclimate and future bioclimatic niche models are likely to be overestimated since they are based solely on projected outcomes, which varies by GCM.

Phylogeographic analyses were well supported and also partially corroborated the hypotheses from the Grinnellian SDM (Figure 3a); the Chiricahua and Utah populations represented separate populations by posterior distribution and maximum likelihood values. However, the Santa Fe population was nested within the Gila, Tonto, and Coconino populations, consistent with the PCA; this may be due to proximity of populations across the landscape or time since population divergence (Bryson et al., 2016). Our results from *F*_ST_ and *Structure* reflect four distinct populations structured across the landscape that are putatively differentiated from each other. These results support change in suitable habitat over time for these populations as indicated by the paleoclimate SDMs, since the Santa Fe population has experienced minimal suitable habitat between it and other populations but is speculated to be recently founded, and the Utah population has ostensibly received migrants more frequently through time (Figure 3b,c).

Overall, these findings indicate that current distributional patterns in *Hetaerina vulnerata* reflect how changes in environmental conditions from the Last Glacial Maximum have geographically and genetically isolated populations. Our results demonstrate that *H. vulnerata* exhibits some structuring and is not panmictic. Indeed, habitat predictors that *H. vulnerata* rely on for oviposition were supported as an isolating variable for this species, especially since these montane riparian ecosystems are surrounded by lowland desert. Projected future environmental conditions suggest continued isolation for this striking species.

## AUTHOR CONTRIBUTIONS

**Austin R. Biddy:** Conceptualization (lead); formal analysis (lead); funding acquisition (equal); methodology (lead); writing – original draft (lead); writing – review and editing (equal). **Joseph D. Manthey:** Conceptualization (supporting); methodology (supporting); resources (lead); writing – review and editing (equal). **Jessica L. Ware:** Conceptualization (supporting); methodology (supporting); writing – review and editing (equal). **Nancy E. McIntyre:** Conceptualization (supporting); data curation (supporting); funding acquisition (equal); supervision (lead); writing – review and editing (equal).

## CONFLICT OF INTEREST STATEMENT

The authors have no conflicts of interest to declare.

### OPEN RESEARCH BADGES

This article has earned an Open Data badge for making publicly available the digitally‐shareable data necessary to reproduce the reported results. The data is available at https://www.ncbi.nlm.nih.gov/biosample?LinkName=bioproject_biosample_all&from_uid=1064155.

## BENEFIT‐SHARING STATEMENT

Benefits generated: Benefits from this research accrue from the sharing of our data and results on public databases as described above.

## Data Availability

Raw ddRADseq sequences for this project have been submitted to NCBI under. BioProject: PRJNA1064155, available at https://www.ncbi.nlm.nih.gov/biosample?LinkName=bioproject_biosample_all&from_uid=1064155. Accessions SAMN39419250 through SAMN39419329 are readily and publicly available to download. Metadata, environmental layers for SDM construction, filtered SNP data in VCF format, and code to run analyses are available in a publicly accessible GitHub repository at https://github.com/arbiddy/Hvulnerata/.

## APPENDIX 1

### 1.1

Variables used in SDM construction.

**Table:**

Variables	Description
Bio1	Annual mean temperature
Bio2	Mean diurnal range (mean of monthly (max temp−min temp))
Bio3	Isothermality (Bio 2/Bio 7) (×100)
Bio4	Temperature seasonality (standard deviation × 100)
Bio5	Max temperature of warmest month
Bio6	Min temperature of coldest month
Bio7	Temperature annual range (Bio 5 – Bio 6)
Bio8	Mean temperature of wettest quarter
Bio9	Mean temperature of driest quarter
Bio10	Mean temperature of warmest quarter
Bio11	Mean temperature of coldest quarter
Bio12	Annual precipitation
Bio13	Precipitation of wettest month
Bio14	Precipitation of driest month
Bio15	Precipitation seasonality (coefficient of variation)
Bio16	Precipitation of wettest quarter
Bio17	Precipitation of driest quarter
Bio18	Precipitation of warmest quarter
Bio19	Precipitation of coldest quarter
Elevation^*^	Digital elevation model
Slope^*^	Slope
Flow direction^*^	Direction of stream drainages
Stream^*^	Intermittent and perennial streams
TCC^*^	Tree canopy coverage

*Note*: An asterisk (^*^) indicates variables used solely in the Grinnellian niche SDM.

## APPENDIX 2

### 2.1

AICc, OR, and AUC reported for each climate scenario. Bioclimatic niche and Grinnellian niche SDMs are included. Feature class abbreviations: “*t*” (threshold) and “*p*” (product). The Bioclimatic niche scenario was projected to other climate scenarios, based on the AICc, OR, and AUC values.

**Table:**

	Feature class	AICc	OR	AUC
Bioclimatic niche	*t*	5149	0.027	0.91
Grinnellian niche	*p*, *t*	4807	0.027	0.96

## APPENDIX 3

### 3.1

Average permutation importance of the variables included in the paleoclimate, current, and future bioclimatic SDMs.

**Table:**

Variables	Paleoclimate	Current	Future
Bio2	3	3.9	4
Bio4	7	7.9	8.6
Bio12	55.5	59.2	54.3
Bio15	17.3	16.8	15.9
Bio17	17.2	12.2	17.3

## APPENDIX 4

### 4.1

Differences in gains and losses of habitat suitability based on projected SDMs minus current SDMs. (a) Current bioclimatic SDM with occurrence points (white dots) and a habitat suitability scale, (b) Last Glacial Maximum (GCM–CCSM‐ESM), (c) Mid‐Holocene (GCM–CCSM‐ESM), (d) Future climate (GCM–IPSL‐CM5A‐LR; SSP – 370), and (e) Future climate (GCM–IPSL‐CM5A‐LR; SSP–585).

## APPENDIX 5

### 5.1

Average permutation importance of the variables included in the Grinnellian SDMs, in decreasing order.

**Table:**

Variables	Grinnellian
Bio16	46.9
Bio14	18.2
Bio15	10.2
Bio5	8.1
TCC	6.8
Stream	5.8
Slope	1.4
Bio3	1.2
Bio2	0.8
Dir	0.6

## APPENDIX 6

### 6.1

Characteristics of the number of loci that passed filtering in Stacks and single SNP per locus for each dataset used.

**Table:**

SNP dataset	# of individuals	# of loci	# of SNPs
All individuals (*Hetaerina americana* and *H. vulnerata*)	80	2080	1787
*H. vulnerata* without Zion, Pinaleño, and *H. americana*	60	9971	9240
*H. vulnerata* without Zion and Pinaleño	58	10,940	10,075

## APPENDIX 7

### 7.1

The percentage of missing data (i.e., genotypes, SNPs, and overall) for the dataset containing 58 *Hetaerina vulnerata* individuals after Stacks and SNPfiltR processing (e.g., depth, minor allele count, etc.) and thresholding.

**Table:**

	Genotype (%)	SNP (%)	Overall (%)	# of SNPs retained
Stacks	60	50	38.48	10,075
SNPfiltR	70	50	50.48	2940

## APPENDIX 8

### 8.1

Dendrogram using a “wald.D2” method and a Euclidean distance for the dataset containing all *Hetaerina* individuals. There is clustering by populations, except for one individual from the Gila population in the adjacent Tonto population.

## APPENDIX 9

### 9.1

Mean *F*_ST_ values per locality sampled, using Nei's (1987) pairwise distance formula on the bottom portion of the matrix. The top part of the matrix is Euclidean distance in decimal degrees between populations. *Hetaerina americana* is included here as an outgroup. Since the *H. americana* individuals were collected from two different locations, geographic distance is not provided.

**Table:**

	Gila	Chiricahua	Santa Fe	Tonto	Coconino	Dixie / Zion	Pinaleño
Gila	—	1.743	3.331	3.572	4.440	6.724	2.003
Chiricahua	0.161	—	4.969	3.092	4.003	6.537	0.970
Santa Fe	0.091	0.227	—	5.106	5.599	6.906	4.797
Tonto	0.004	0.179	0.097	—	0.918	3.449	2.122
Coconino	0.007	0.179	0.088	0.003	—	2.559	3.034
Dixie/Zion	0.096	0.225	0.123	0.090	0.079	—	5.567
Pinaleño	0.016	0.112	0.049	0.013	0.015	0.099	—
*H. americana*	0.9041	0.883	0.907	0.893	0.903	0.891	0.847

## APPENDIX 10

### 10.1

(a) PCA and (b) structure plots (*K* = 2–4) after SNPfiltR processing. Colors correspond to Figure 5 for population assignment consistency.

## APPENDIX 11

### 11.1

Maximum likelihood population effects (MPLE) model statistics: AIC and BIC. The models tested were Grinnellian niche and bioclimatic niche.

**Table:**

	AIC	BIC
Grinnellian	12.488	20.277
Bioclimatic	20.459	26.832

## APPENDIX 12

### 12.1

Upper and lower confidence intervals (CI) for the MLPE Grinnellian niche model. CIs that remain negative indicate that they have a strong impact on gene flow, whereas CIs that overlap 0 have a negligible effect on gene flow. See Appendix 1 for description of variables.

**Table:**

	2.5% CI	97.5% CI
Intercept	−4.726	11.142
Bio2	−0.628	0.391
Bio8	−0.204	0.065
Bio9	−0.117	0.101
Bio15	−0.042	0.064
Direction	−0.007	0.089
Stream	−2.17	3.317
Elevation	−0.003	0.001
TCC	−0.145	−0.052

## APPENDIX 13

### 13.1

Upper and lower confidence intervals (CI) for the MLPE bioclimatic niche model. CIs that remain negative indicate that they have a strong impact on gene flow, whereas CIs that overlap 0 have a negligible effect on gene flow. See Appendix 1 for description of variables.

**Table:**

	2.5% CI	97.5% CI
Intercept	−6.984	10.75
Bio2	−0.409	0.300
Bio8	−0.149	0.134
Bio9	−0.018	0.151
Bio17	−0.084	0.040
Bio18	−0.019	0.004
Bio19	−0.015	0.005

## APPENDIX 14

### 14.1

Tree canopy coverage percentage for the study extent. Dark green colors indicate high tree canopy coverage, and sampled populations are represented by the pink dots.
